# Calorimetrically Calibrated Ultrasound-Assisted Fermentation Accelerates Isoflavone Aglycone Biotransformation in *Astragalus membranaceus*

**DOI:** 10.3390/foods15213909

**Published:** 2026-10-31

**Authors:** Zhihao Zheng, Tao Chen, Haohan Zhao, Jikang Chen, Shuai Song, Jinwen Cai, Haixuan Wang, Jie Luo, Maiquan Li, Yingjun Zhou, Xiaochun Li, Li Yan, Yuande Peng, Chunliang Xie

**Affiliations:** 1 Institute of Bast Fiber Crops, Chinese Academy of Agricultural Sciences, Changsha 410205, China; 2 Yuelushan Laboratory, Changsha 410128, China; 3 Jiangsu FTCM Life Science and Technology Development Co., Ltd., Yancheng 224100, China; 4 College of Food Science and Technology, Hunan Agricultural University, Changsha 410128, China

**Keywords:** ultrasound-assisted fermentation, *Astragalus membranaceus*, *Lactiplantibacillus plantarum*, β-glucosidase, isoflavone aglycones, calorimetric calibration

## Abstract

Ultrasound-assisted fermentation (UAF) was evaluated as a calorimetrically calibrated strategy for biotransforming *Astragalus membranaceus* isoflavones using *Lactiplantibacillus plantarum*. Water-based calorimetry yielded power-density estimates of 46.51 ± 4.03 and 86.05 ± 4.03 W/L for the fermentation vessel configuration. High-dose UAF accelerated the conversion of isoflavone glucosides into calycosin and formononetin, reaching 99.7 ± 2.4% of the conventional 24 h aglycone level by 18 h with a 50% higher mean formation rate over 10–18 h. No aglycone formation was detected in the sterile ultrasound control under the tested conditions. At the 18 h time-matched sampling point, high-dose UAF yielded higher in vitro antioxidant capacities, stronger DPP-IV and pancreatic lipase inhibition, and greater measured intestinal-phase calycosin recovery (374.80 vs. 314.31 μg/g) than conventional fermentation. At 16 h, extracellular β-glucosidase activity increased dose-dependently from 3.52 ± 0.82 to 11.19 ± 0.45 U/mL (conventional vs. high-dose UAF), whereas intracellular activity remained comparable across treatments. Combined with qualitative SEM and CLSM observations, these findings are consistent with enhanced extracellular enzyme availability and plant matrix disruption, without establishing de novo enzyme synthesis or a specific permeabilisation mechanism.

## 1. Introduction

Plant-derived, isoflavone-rich matrices are increasingly incorporated into functional fermented beverages, postbiotic formulations, and plant-protein-fortified food products. Among them, the rhizome of *Astragalus membranaceus* has attracted attention as a food-compatible botanical ingredient because its aqueous extracts contain calycosin-7-*O*-glucoside, ononin, and their corresponding aglycones, calycosin and formononetin [1]. *Astragalus membranaceus* has been officially designated as a medicine-and-food homologous material in China and is commercially used in functional beverages and fermented food products [2,3]. From a food-processing perspective, the central question is not simply whether *Astragalus* contains bioactive compounds, but which chemical forms predominate after processing and what quantities are recovered following simulated gastrointestinal digestion.

In native rhizome material and conventional aqueous extracts, calycosin and formononetin occur largely as β-D-glucoside conjugates. Whereas the glycosylated forms retain a polar sugar moiety, deglycosylation exposes the phenolic scaffold more directly in the corresponding aglycones. Consequently, the primary target of fermentation is structural biotransformation rather than a net increase in total isoflavone mass [4]: the process shifts a glucoside-rich profile toward bioactive aglycones, with the resulting composition and functionality evaluated by targeted HPLC and in vitro food-matrix assays.

Lactic acid bacterial fermentation can mediate glycoside-to-aglycone biotransformation in fermented food matrices [5]. *Lactiplantibacillus plantarum* can express β-glucosidase activity that hydrolyses β-glycosidic bonds in plant glucosides, and this activity is relevant to glycoside biotransformation during food fermentation [6,7]. β-Glucosidase localisation can influence substrate accessibility; cell-surface localisation has been reported for an individual *L. plantarum* enzyme [8] but has not been established for XD087. In the present system, an enzyme–matrix interface is therefore considered a possible explanation.

Ultrasound is widely applied in food processing for extraction, dispersion, microbial inactivation, cell permeabilisation, and mass-transfer enhancement [9,10]. Its incorporation into fermentation—ultrasound-assisted fermentation (UAF)—has been examined across dairy, soybean, and herbal systems, with reported effects ranging from accelerated microbial growth and a shortened lag phase to enhanced metabolite release [11,12]. A recurring limitation in the UAF literature, however, is the conflation of nominal instrument power with the acoustic dose actually transmitted into the sample [13]. This discrepancy is particularly pronounced for indirect bath-type ultrasound, in which most of the electrical input is dissipated into the bath water, transducer assembly, and vessel wall, leaving only a small fraction to reach the sample. Without calorimetric calibration performed under the same sample volume, vessel geometry, bath level, and flask position used during fermentation, nominal bath power-density ratings provide limited inter-laboratory comparability. Process reproducibility, dose–response interpretation, and mechanistic evaluation all depend on this calibration, which remains under-reported in food-fermentation UAF studies [14].

Mechanistic interpretations of UAF require evidence that distinguishes altered enzyme availability from altered enzyme synthesis. The present study did not measure bacterial gene expression; therefore, it does not claim changes in β-glucosidase-related transcription. It examines whether calibrated ultrasound is associated with increased extracellular β-glucosidase availability and physical changes in the *Astragalus* matrix without invoking unmeasured synthesis. Earlier ultrasound-fermentation studies reported increased β-glucosidase activity without resolving its origin [14,15]. Here, extracellular and intracellular activity were measured in parallel, allowing the observed pattern to be interpreted in relation to enzyme availability.

Accordingly, this study evaluated calorimetrically calibrated UAF during logarithmic-phase *L. plantarum* fermentation of *Astragalus*. Under water-calibrated ultrasound settings, we examined: (i) whether ultrasound shortens the fermentation time required to reach the 24 h aglycone concentration of conventional fermentation; (ii) whether this acceleration is accompanied by increased extracellular β-glucosidase availability without significant differences in intracellular activity; and (iii) whether ultrasound alone induces direct sonochemical hydrolysis in a sterile control. Process performance and matrix properties were characterised using targeted HPLC, partitioned enzyme activity assays, SEM and CLSM imaging, in vitro antioxidant and digestive-enzyme inhibitory assays, and static simulated gastrointestinal digestion.

## 2. Materials and Methods

### 2.1. Materials and Reagents

Dried rhizomes of *Astragalus membranaceus* were obtained from Bozhou Fushen Pharmaceutical Co., Ltd. (Bozhou, China). Reference standards of calycosin-7-*O*-β-D-glucoside, calycosin, ononin, and formononetin (purity > 98%) were purchased from Shanghai Yuanye Bio-Technology Co., Ltd. (Shanghai, China). A β-glucosidase activity assay kit (colourimetric method) as well as pepsin (porcine gastric mucosa), pancreatin (porcine pancreas), and bile salts for in vitro simulated digestion were purchased from Beijing Solarbio Science & Technology Co., Ltd. (Beijing, China). HPLC-grade acetonitrile and methanol were obtained from TEDIA (Fairfield, OH, USA). All other reagents were of analytical grade and obtained from commercial suppliers.

### 2.2. Microbial Activation and Astragalus Medium Preparation

*Lactiplantibacillus plantarum* XD087 was deposited at the Agricultural Product Bioprocessing Team, Institute of Bast Fiber Crops, Chinese Academy of Agricultural Sciences. The strain was cryopreserved at −80 °C in MRS broth supplemented with 25% (*v*/*v*) glycerol and activated through two successive 10 h subcultures in sterile MRS broth at 37 °C. An activated culture at OD_600_ ≈ 1.0, corresponding to approximately 1 × 10^8^ CFU/mL, was used as the inoculum [16].

Dried *Astragalus* rhizomes were pulverised and passed through an 80-mesh sieve. The resulting powder was suspended in distilled water at a 1:10 (*w*/*v*) ratio and sterilised at 115 °C for 15 min to obtain the *Astragalus* fermentation medium.

### 2.3. Calorimetric Calibration of Ultrasound Settings

Fermentation was conducted in 100 mL Erlenmeyer flasks containing 30 mL of *Astragalus* medium. Ultrasound was applied using a KQ-500DB digital ultrasonic bath (Kunshan Ultrasonic Instrument Co., Ltd., Kunshan, China; 40 kHz; rated total bath power, 500 W). The bath contained 4.97 L of water; with the 100 mL Erlenmeyer flask (30 mL sample) in place, the total bath load was approximately 5.00 L, such that the 100 W/L nominal condition corresponded to the 500 W rated bath power. Because indirect bath-type ultrasound transmits only a fraction of the nominal bath input into the sample, water-based calorimetric estimates were obtained under the exact vessel, sample volume, bath level, and flask position used during fermentation [17].

A 30.0 mL volume of deionised water (initial temperature 27–31 °C) was sonicated for 600 s at each nominal bath power-density setting, and temperatures before and after sonication were recorded with a calibrated thermocouple immersed in the centre of the flask. Acoustic power was calculated from the mass and specific heat capacity of water without correcting for vessel heat capacity or heat exchange with the bath and surroundings; accordingly, the resulting values represent configuration-specific water-calorimetric estimates rather than fully corrected absorbed power measurements in the fermentation matrix. Power (*P*) was estimated as:

*P* = *m* × *C*_p_ × Δ*T*/*t*

where *m* is the water mass (0.030 kg), *C*_p_ is the specific heat capacity of water (4186 J kg^−1^ K^−1^), Δ*T* is the temperature increase, and *t* is the sonication time. The estimated power density was calculated as *P*/0.030 L. Three independent replicates were performed at each setting.

### 2.4. Ultrasound-Assisted Fermentation Design

Preliminary experiments showed that applying ultrasound at 10 h, corresponding to the mid-logarithmic growth phase under the present fermentation conditions, provided the most favourable balance between growth maintenance and enzyme externalisation (Figures S1 and S2). Five experimental groups were therefore established under the water-calibrated ultrasound settings:

- G1—conventional fermentation: 2% (*v*/*v*) *L. plantarum* XD087, 37 °C, no ultrasound.
- G2—low-dose UAF: 2% (*v*/*v*) *L. plantarum* XD087, ultrasound at 46.51 ± 4.03 W/L for 20 min applied at 10 h of fermentation.
- G3—high-dose UAF: 2% (*v*/*v*) *L. plantarum* XD087, ultrasound at 86.05 ± 4.03 W/L for 20 min applied at 10 h of fermentation.
- G4—sterile + ultrasound control: uninoculated sterile *Astragalus* medium exposed to the same 86.05 ± 4.03 W/L treatment as G3, included to test whether ultrasound alone could hydrolyse the target isoflavone glucosides in the absence of microbial enzymes.
- G5—sterile, untreated blank.

All fermentations were maintained for 72 h.

### 2.5. Bacterial Growth and pH Determination

Samples were collected at 0, 4, 8, 10, 10.5, 16, 24, 36, 48, 60, and 72 h. Bacterial growth was monitored by standard plate counting on MRS agar. The specific growth rate (*μ*) was calculated from viable counts as *μ* = (ln *N*_2_ − ln *N*_1_)/(*t*_2_ − *t*_1_), where *N*_1_ and *N*_2_ are the colony counts (CFU mL^−1^) at times *t*_1_ and *t*_2_. To evaluate the effect of the 10 h ultrasound intervention, *μ* was determined across the 0–16 h growth window as well as separately for the pre-treatment (0–10 h) and post-treatment (10–16 h) sub-intervals. The pH of the fermentation broth was measured with a SevenExcellence S500-Basic precision pH meter (Mettler Toledo, Columbus, OH, USA).

### 2.6. HPLC Quantification of Isoflavones

Fermentation samples collected at 10, 13, 14, 15, 16, 17, 18, 19, 20, and 24 h were extracted with 80% methanol (1:10, *w*/*v*), centrifuged, and filtered through a 0.22 μm membrane [18]. Calycosin-7-*O*-glucoside, calycosin, ononin, and formononetin were quantified using a Shimadzu SIL-16 HPLC system equipped with a C18 column (4.6 mm × 250 mm, 5 μm). Mobile phase A was acetonitrile, and mobile phase B was 0.1% aqueous phosphoric acid. The column temperature was 30 °C, the injection volume was 10 μL, and the detection wavelength was 260 nm. Gradient elution was programmed as follows: 0–5 min, 20% A; 5–20 min, 20–40% A; 20–30 min, 40% A; 30–40 min, 40–20% A; and 40–45 min, 20% A. Calibration curves of the four reference standards were linear across the working range (*R*^2^ > 0.999).

### 2.7. In Vitro Antioxidant and Food-Matrix Digestive-Enzyme Assays

Following modified colourimetric methods [19,20,21,22,23], samples fermented for 18 h were assayed for hydroxyl radical (·OH) scavenging activity, reducing power (Prussian blue method), ABTS·^+^ radical scavenging activity, α-glucosidase inhibition, dipeptidyl peptidase-IV (DPP-IV) inhibition, and pancreatic lipase inhibition. For the enzyme inhibitory assays, the inhibition rate was calculated as:

Inhibition (%) = [1 − (A*sample* − A*sample blank*)/(A*control* − A*control blank*)] × 100

For the ·OH and ABTS·^+^ assays, the scavenging rate was calculated as:

R(%) = (1 − A*sample*/A*control*) × 100

### 2.8. In Vitro Simulated Gastrointestinal Digestion

Static in vitro simulated gastrointestinal digestion was performed following the INFOGEST consensus protocol with modified parameters adapted from published studies [24,25]. Simulated salivary fluid (SSF), simulated gastric fluid (SGF), and simulated intestinal fluid (SIF) were prepared accordingly. The nominal enzyme activities specified by the supplier were as follows: pepsin, 1:3000–1:3500 (digestion ratio); pancreatin trypsin activity ≥ 250 N.F.U/mg; and α-amylase, 3700 U/g. Freeze-dried powder (0.50 g) from 18 h fermentation samples was mixed with 5.00 mL of deionised water and 0.50 mL of SSF and shaken at 37 °C and 100 rpm for 5 min, after which salivary enzymes were inactivated by boiling for 5 min. After cooling and adjusting the pH to 2.0 with 6 M HCl, 7.5 mL of SGF was added, and the mixture was shaken at 37 °C and 120 rpm for 120 min. Gastric enzymes were then inactivated by heating, and the pH was adjusted to 7.6 with 4 M NaOH. Subsequently, 7.5 mL of SIF was added, and the mixture was shaken at 37 °C and 120 rpm for 120 min, followed by final thermal inactivation.

Following digestion, a post-digestion ultrasonication step at 50 °C was applied identically to all groups for analytical homogenisation immediately before colourimetric or HPLC analysis; thus, the reported digestion endpoints reflect recovery after this shared sample-preparation step.

Total phenolics in the intestinal-phase digesta were determined by the Folin–Ciocalteu method using gallic acid as the standard and expressed as mg gallic acid equivalents per gram of dry sample (mg GAE/g) [26]. Total flavonoids were determined by the aluminium nitrate colourimetric method using rutin as the standard and expressed as mg rutin equivalents per gram of dry sample (mg RE/g) [27]. Calycosin and formononetin in the intestinal-phase digesta were quantified by HPLC as described in Section 2.6.

Measured intestinal-phase recovery was expressed as the analyte amount quantified in the processed digesta per gram of initial dry sample (μg/g, mg GAE/g, or mg RE/g, as applicable), representing absolute mass recovery after the shared post-digestion homogenisation step rather than percentage bioaccessibility or a separately isolated micellar fraction.

### 2.9. Intracellular and Extracellular β-Glucosidase Partitioning

To examine whether UAF increased extracellular enzyme availability without altering intracellular enzyme levels, β-glucosidase activity was partitioned into extracellular and intracellular fractions across the ultrasound intervention window (10, 10.5, 13, 16, 20, and 24 h). The fermentation broth was centrifuged at 8000× *g* for 10 min at 4 °C, and the supernatant was collected as the extracellular fraction. The cell pellet was washed twice with ice-cold PBS, resuspended in PBS, disrupted by sonication on ice (3 s pulse, 3 s rest, 5 min total at low amplitude to avoid heating), and centrifuged at 12,000× *g* for 10 min at 4 °C; the resulting supernatant was collected as the intracellular fraction. β-Glucosidase activity in both fractions was measured under identical assay conditions.

### 2.10. Morphological Characterisation (SEM and CLSM)

To visualise the microstructural impact of UAF on the *Astragalus* matrix, samples fermented for 18 h were freeze-dried, gold-sputtered, and examined by scanning electron microscopy (SEM; Phenom Pure, ProX G6, Thermo Fisher, Waltham, MA, USA) at 5 kV and 1000× magnification.

To visualise the impact of UAF on bacterial cell-envelope integrity, ultrasound-treated *L. plantarum* cells were stained using a LIVE/DEAD BacLight Bacterial Viability Kit (Thermo Fisher) according to the manufacturer’s instructions and imaged by confocal laser scanning microscopy (LSM 900, Zeiss, Oberkochen, Germany) using SYTO9 (green; nucleic-acid counterstain) and propidium iodide (PI; red; selective for membrane-compromised cells).

### 2.11. Statistical Analysis

All experiments were performed in triplicate unless otherwise stated, and data are presented as mean ± standard deviation. Group differences in the biochemical endpoints (isoflavone contents, β-glucosidase activities, antioxidant capacities, enzyme-inhibition rates, and measured intestinal-phase recovery values) were analysed by one-way ANOVA followed by Tukey’s post hoc test (GraphPad Prism, version 8.0.2), with statistical significance set at *p* < 0.05. Specific growth rates (*μ*), derived from triplicate viable counts, are reported as mean ± standard deviation and compared descriptively as numerical trends.

## 3. Results and Discussion

### 3.1. Calorimetric Calibration Characterises Ultrasound Settings Under the Fermentation Vessel Configuration

Calorimetric calibration revealed that the nominal bath power density alone did not reflect the actual acoustic energy absorbed by the 30 mL fermentation sample. At nominal bath power-density settings of 50 and 100 W/L, the calibration yielded power estimates of 1.40 ± 0.12 and 2.58 ± 0.12 W in the 30 mL flask, corresponding to water-calibrated power-density estimates of 46.51 ± 4.03 and 86.05 ± 4.03 W/L, respectively (Table 1). These estimates define the acoustic output of the two ultrasound settings under the specific vessel configuration. This consideration is particularly critical for indirect bath systems, where energy transfer depends heavily on bath load, vessel geometry, acoustic coupling, and flask position rather than nominal instrument ratings alone [28].

Although this water calibration reproduced the exact vessel, sample volume, bath level, and flask position used during fermentation, the endpoint temperature-rise method does not correct for vessel heat capacity or ambient heat exchange (Section 2.3); accordingly, these values serve as configuration-specific reference estimates.

### 3.2. Calibrated Ultrasound Preserves L. plantarum Growth and Metabolic Acidification

**Figure 1:**
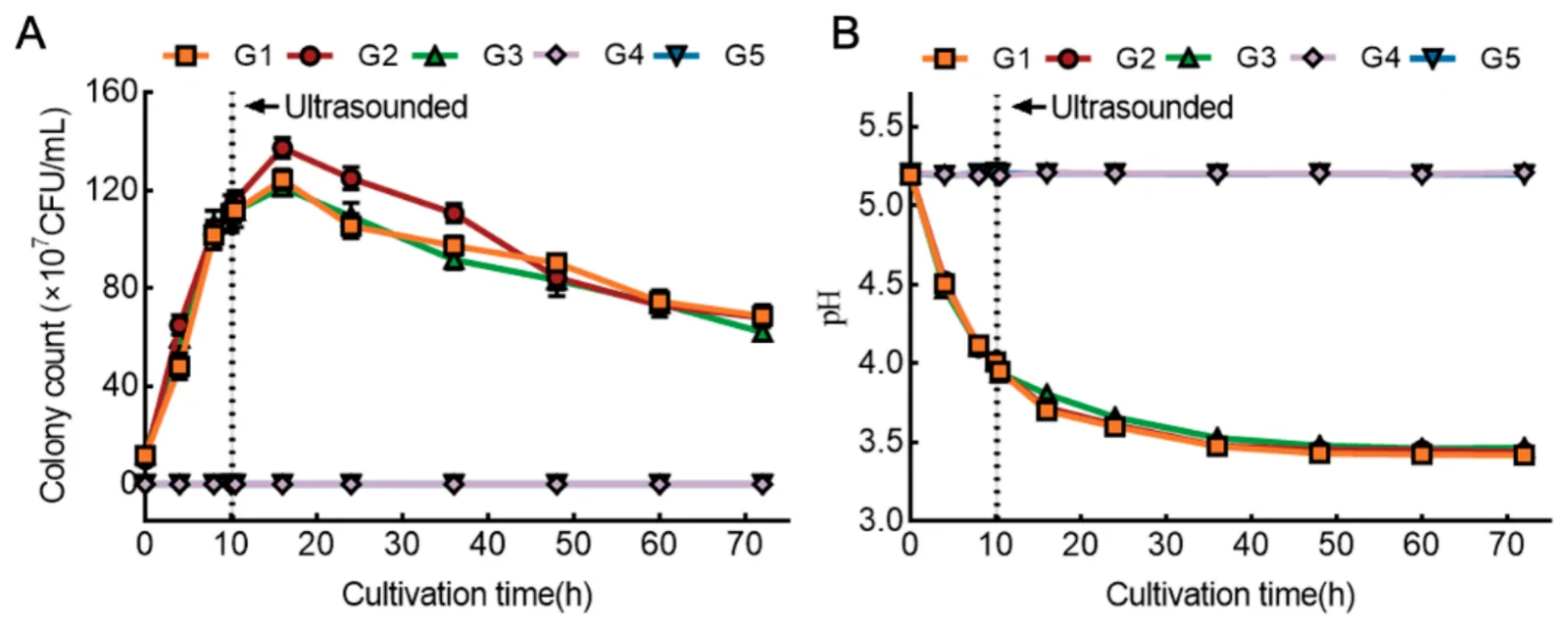
Effects of calibrated ultrasound on growth kinetics and metabolic acidification during *Astragalus* fermentation by *L. plantarum*. (**A**) Viable cell counts under different water-calibrated ultrasound settings. (**B**) Broth pH dynamics. G1, conventional fermentation; G2, low-dose UAF (46.51 ± 4.03 W/L); G3, high-dose UAF (86.05 ± 4.03 W/L); G4, sterile + high-dose ultrasound; G5, sterile blank. Data are mean ± SD (*n* = 3). The dashed line indicates ultrasound treatment at 10 h. All inoculated groups maintained typical lactic acid fermentation kinetics, indicating that the ultrasound intervention operated within an active culture rather than acting as a lethal extraction step.

Under the calibrated doses, viable cell counts in all inoculated groups (G1, G2, and G3) increased steadily through the logarithmic phase and reached a stationary plateau (Figure 1A). Prior to ultrasound intervention (0–10 h), the three groups exhibited comparable specific growth rates (G1, 0.2261 ± 0.0175 h^−1^; G2, 0.2423 ± 0.0324 h^−1^; G3, 0.2227 ± 0.0052 h^−1^). Across the 0–16 h growth window, G2 showed the highest numerical specific growth rate (*μ* = 0.1657 ± 0.0193 h^−1^), followed by G1 (*μ* = 0.1489 ± 0.0160 h^−1^) and G3 (*μ* = 0.1447 ± 0.0041 h^−1^). A similar numerical pattern appeared during the post-treatment window (10–16 h: G2, 0.0381 ± 0.0035 h^−1^; G1, 0.0201 ± 0.0153 h^−1^; G3, 0.0149 ± 0.0045 h^−1^). Differences in numerical specific growth rate across treatments were not statistically significant, especially for G1 during the post-treatment period, indicating that neither ultrasound dose impaired microbial growth. As expected, no growth was observed in uninoculated controls (G4 and G5) [11].

Metabolic acidification was similarly preserved: the pH of all inoculated groups declined from approximately 6.5 to 4.0 during fermentation (Figure 1B), whereas the uninoculated G4 and G5 controls remained near neutral. Together, these results confirm sustained lactic acid fermentation under both ultrasound doses [14].

### 3.3. Calibrated UAF Accelerates Isoflavone Aglycone Biotransformation and Shortens Fermentation Time

**Figure 2:**
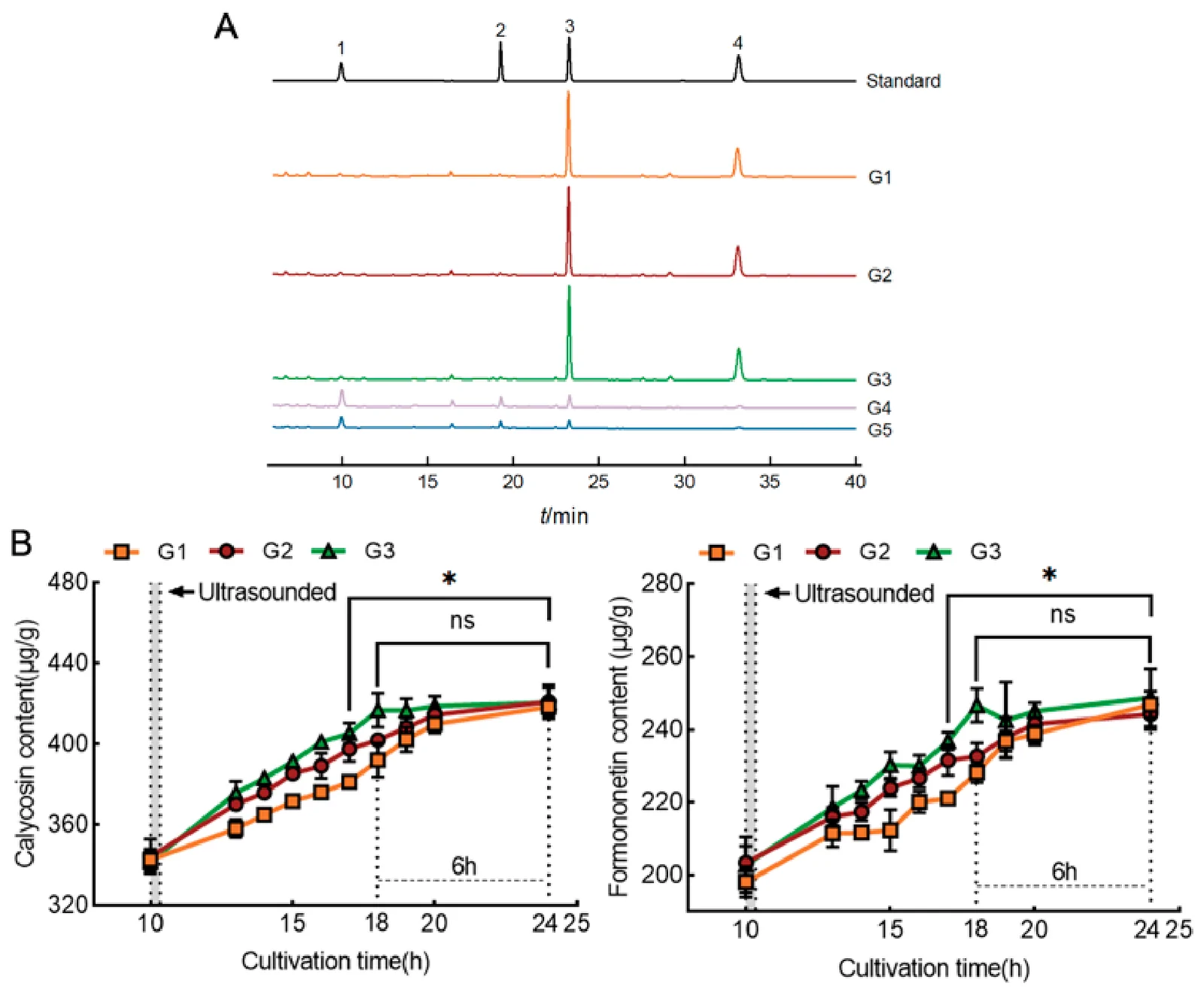
UAF accelerates the biotransformation of *Astragalus* isoflavones. (**A**) Representative HPLC chromatograms at 18 h: peak 1, calycosin-7-*O*-glucoside; peak 2, ononin; peak 3, calycosin; peak 4, formononetin. (**B**) Time courses of calycosin and formononetin concentrations from 10 to 24 h. The grey dashed vertical line indicates the time point of ultrasound application (10 h). At 18 h, high-dose UAF (G3) reached 99.7 ± 2.4% of the total aglycone concentration measured in G1 at 24 h (Table 2), reflecting earlier attainment of that reference level. * indicates significant difference (*p* < 0.05); ns indicates no significant difference. The sterile + ultrasound control (G4) showed no detectable aglycone formation, providing no evidence of direct sonochemical hydrolysis of the target glucosides under the tested conditions.

Targeted HPLC time-course profiling revealed the progression of isoflavone biotransformation following the calibrated UAF treatment (Figure 2). Across the 10–24 h window, calycosin-7-*O*-glucoside (peak 1) and ononin (peak 2) progressively declined while calycosin (peak 3) and formononetin (peak 4) accumulated concurrently, consistent with the enzymatic cleavage of β-glycosidic bonds [4,29]. In G1, calycosin and formononetin reached their maxima at 24 h, whereas G3 attained 99.7% of the G1 24 h aglycone level by 18 h (Table 2). The primary benefit of calibrated UAF is a 6 h (25%) reduction in the fermentation time needed to achieve equivalent aglycone profiles. 

Kinetic parameters derived from the concentration–time profiles further substantiate this acceleration (Table 2). During 10–18 h, the mean total aglycone formation rate in G3 was 14.87 ± 3.11 μg g^−1^ h^−1^, 49.8% higher than G1 (9.93 ± 2.37 μg g^−1^ h^−1^). By 18 h, G3 had reached 99.7 ± 2.4% of the total aglycone concentration measured in G1 at 24 h (663.7 vs. 665.6 μg g^−1^). Between 18 and 24 h, total aglycones in G3 increased by only 0.9 ± 4.1%, compared with 7.3 ± 2.0% in G1. Over 10–24 h, integrated concentration–time areas (AUC_10–24h_) differed by less than 4% among groups, and 24 h total aglycone concentrations converged at 665.6 ± 7.2, 665.5 ± 12.6, and 669.6 ± 14.8 μg g^−1^ in G1, G2, and G3, respectively. Together, these kinetic metrics support faster aglycone accumulation toward the conventional reference concentration.

To evaluate whether physical sonochemistry alone contributed to deglycosylation, the sterile + ultrasound control (G4) was exposed to the same 86.05 ± 4.03 W/L treatment as G3 without microbial inoculation. No detectable formation of calycosin or formononetin occurred in G4 (Figure 2A), providing no evidence of direct ultrasonic hydrolysis of calycosin-7-*O*-glucoside or ononin under these matrix conditions. Aglycone accumulation in G2 and G3 therefore required microbial fermentation, consistent with ultrasound facilitating the fermentation interface. The enzymatic and microstructural mechanisms underlying this accelerated aglycone release in G3 are discussed in Section 3.7.

### 3.4. Calibrated UAF Improves In Vitro Antioxidant Activity of the Astragalus Matrix

**Figure 3:**
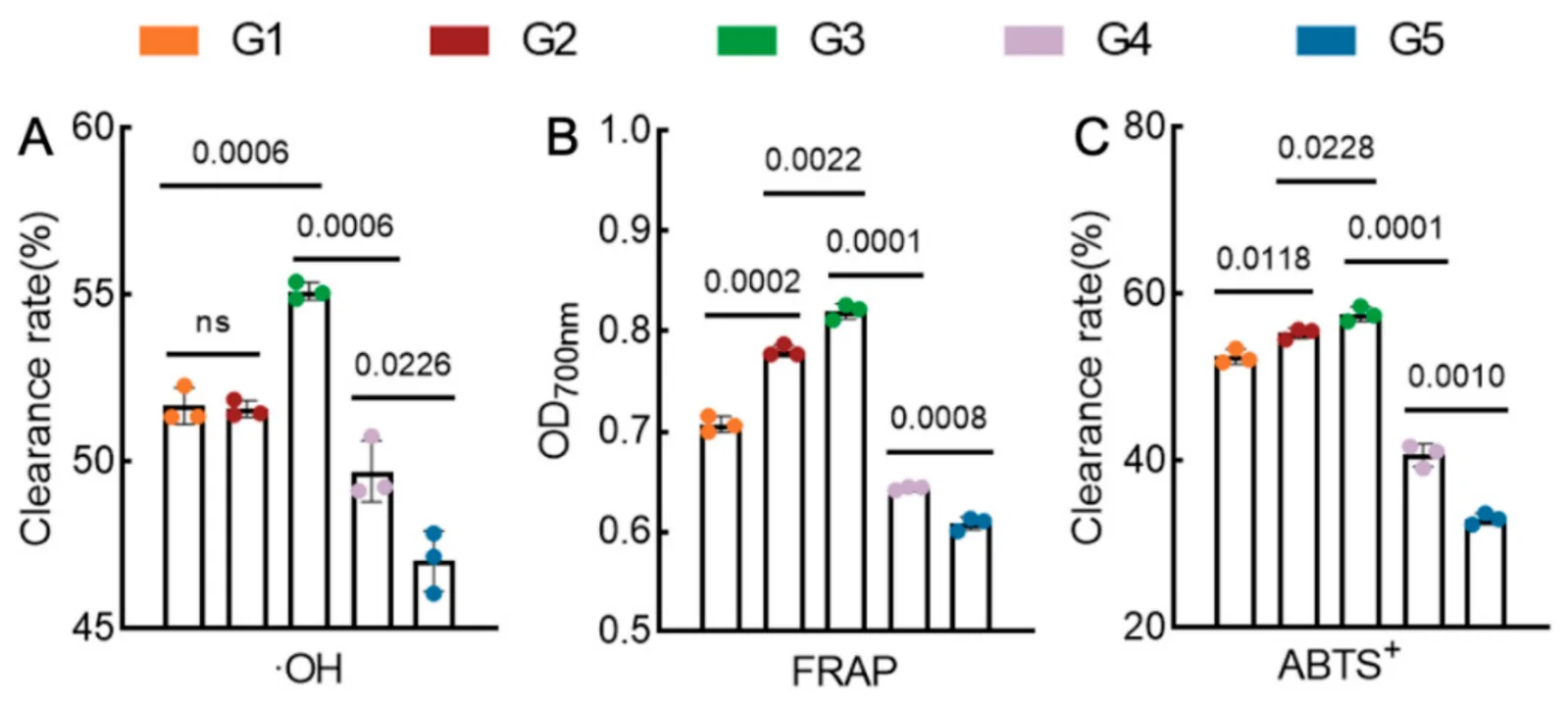
In vitro antioxidant activities of *Astragalus* fermentation samples at 18 h: (**A**) hydroxyl radical scavenging activity, (**B**) reducing power (Prussian blue method), and (**C**) ABTS·^+^ radical scavenging activity. G3 significantly outperformed G1 across all three indicators (*p* < 0.05), whereas the G4 and G5 controls showed minimal activity. ns indicates no significant difference.

Because the high-dose UAF group (G3) achieved ~99.7% of the conventional 24 h aglycone yield by 18 h (Table 2), this time point was selected as the standardized sampling benchmark for functional evaluations. Consequently, the 24 h functional profile of G1 was omitted from this comparison. At 18 h, hydroxyl radical scavenging activity in G3 reached 55.12%, reducing power (Prussian blue method, absorbance) reached 0.82, and ABTS·^+^ radical scavenging activity reached 57.62%, each significantly higher than the corresponding value in G1 (*p* < 0.05; Figure 3). G4 and G5 showed minimal activity.

Chemically, this enhancement aligns with the glucoside-to-aglycone conversion shown in Figure 2. Deglycosylation liberates free phenolic hydroxyl groups on calycosin and formononetin, facilitating the hydrogen-atom-transfer (HAT) and single-electron-transfer (SET) reactions that govern the antioxidant assays, whereas steric and electronic masking by the glycosyl moiety attenuates the activity of their parent glucosides [4,30]. These results indicate that the enhanced antioxidant capacity stems from aglycone enrichment rather than de novo synthesis of novel bioactive compounds by ultrasound.

### 3.5. Calibrated UAF Enhances In Vitro Enzyme Modulation

**Figure 4:**
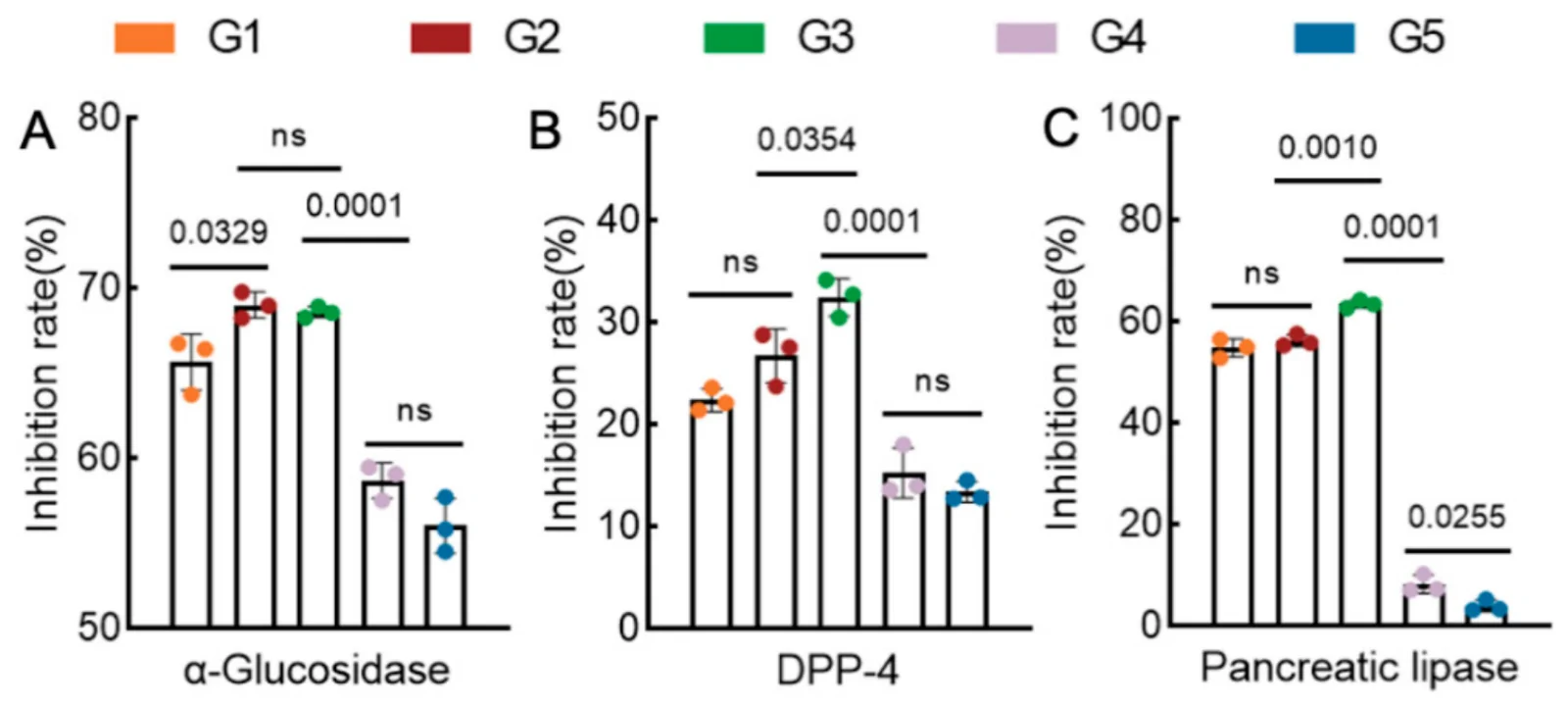
*In vitro* digestive-enzyme inhibitory activities of fermented *Astragalus* extracts at 18 h against (**A**) α-glucosidase, (**B**) DPP-IV, and (**C**) pancreatic lipase. G3 exhibited inhibition rates of 68.63%, 32.53%, and 63.59%, respectively, each significantly higher than that of G1 (*p* < 0.05). ns indicates no significant difference.

In addition to antioxidant activity, *in vitro* inhibitory assays against α-glucosidase, dipeptidyl peptidase-IV (DPP-IV), and pancreatic lipase were performed at 18 h to evaluate the metabolic-regulatory potential of the fermented matrix. Under high-dose UAF (G3), the fermented *Astragalus* extract inhibited α-glucosidase, DPP-IV, and pancreatic lipase by 68.63%, 32.53%, and 63.59%, respectively, all significantly higher than the conventionally fermented control (G1; *p* < 0.05; Figure 4). These results indicate that accelerated aglycone enrichment was accompanied by stronger *in vitro* digestive-enzyme inhibition [31,32].

### 3.6. Calibrated UAF Increases Measured Intestinal-Phase Recovery of Isoflavones, Total Phenolics and Total Flavonoids

Bioactive recovery following gastrointestinal transit was evaluated using a static *in vitro* simulated digestion model. Samples were collected at 18 h across all groups, and intestinal-phase recovery was quantified after the analytical homogenisation step described in Section 2.8. 

**Figure 5:**
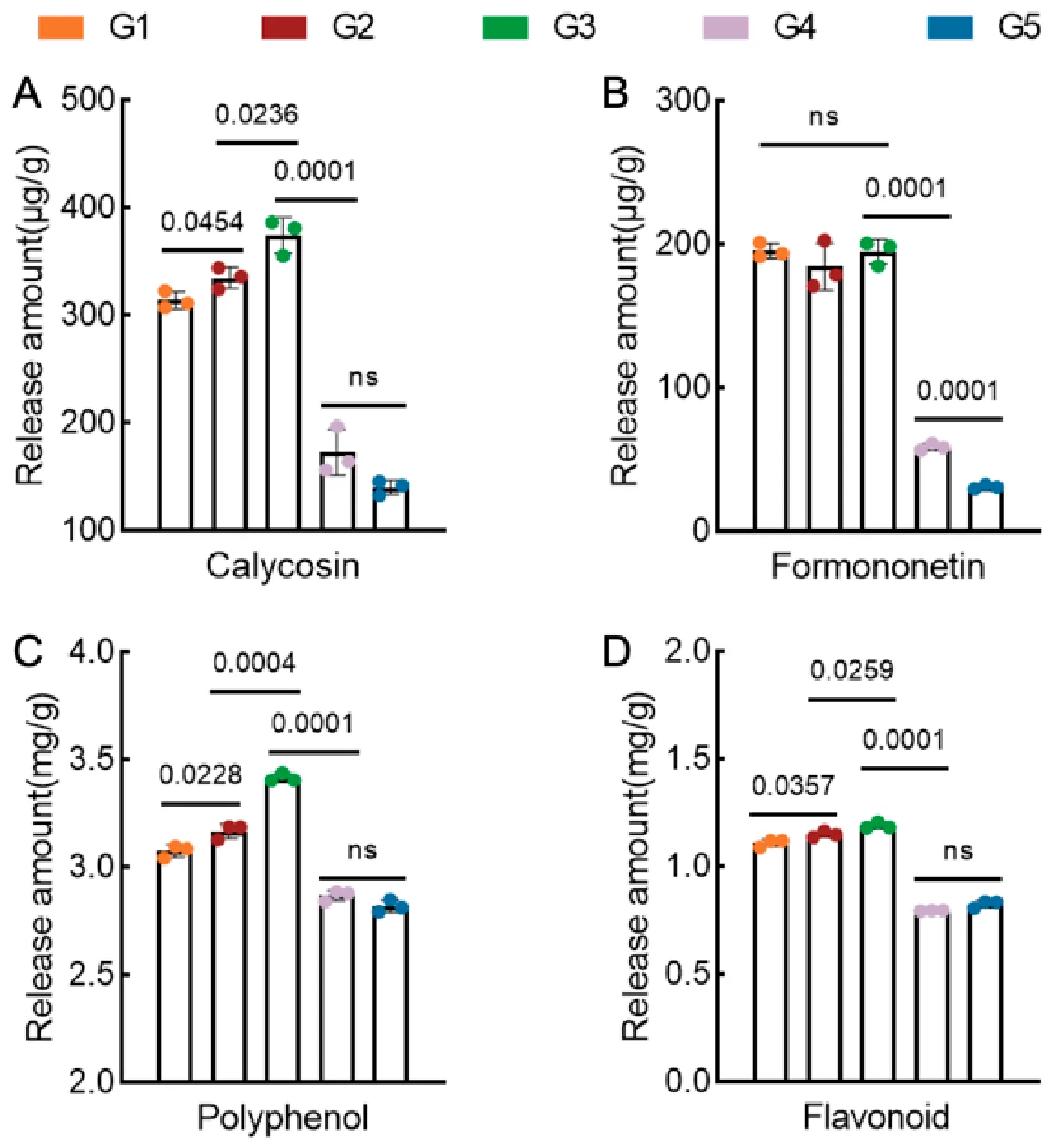
Intestinal-phase recovery of bioactive components after in vitro simulated gastrointestinal digestion: (**A**) calycosin recovery (μg/g dry sample), (**B**) formononetin recovery (μg/g dry sample), (**C**) total phenolics (mg GAE/g), and (**D**) total flavonoids (mg RE/g). G3 showed the highest measured amounts across all four indicators, with calycosin rising from 314.31 μg/g (G1) to 374.80 μg/g (G3). ns indicates no significant difference between groups; values above the bars represent the *p*-values of corresponding intergroup comparisons. Post-digestion ultrasonication at 50 °C was applied identically to all groups for analytical homogenisation; the axis label “Release amount” denotes measured intestinal-phase recovery normalised to the initial dry sample mass rather than fractional bioaccessibility.

Measured intestinal-phase calycosin recovery in G3 reached 374.80 μg/g, compared with 314.31 μg/g in G1 (Figure 5A), with formononetin recovery showing a corresponding increase (Figure 5B). Total phenolics and total flavonoids in the intestinal-phase digesta of G3 were also significantly higher than those in G1 (Figure 5C,D; total phenolics, 3.41 vs. 3.08 mg GAE/g; total flavonoids, 1.19 vs. 1.11 mg RE/g). G2 exhibited intermediate values between G1 and G3 across all four indicators, consistent with the dose-dependent patterns observed in Figure 2 and Figure 3.

Two possible explanations are a higher aglycone-to-glucoside ratio in G3 and the matrix changes observed by SEM (Section 3.7.2). Differences in initial analyte content and matrix-dependent recovery during the shared analytical processing may also contribute to the measured intestinal-phase amounts [30,33].

### 3.7. Evidence Consistent with a Dual-Interface Explanation for Accelerated Biotransformation

#### 3.7.1. Extracellular β-Glucosidase Availability Without Evidence of De Novo Synthesis

**Figure 6:**
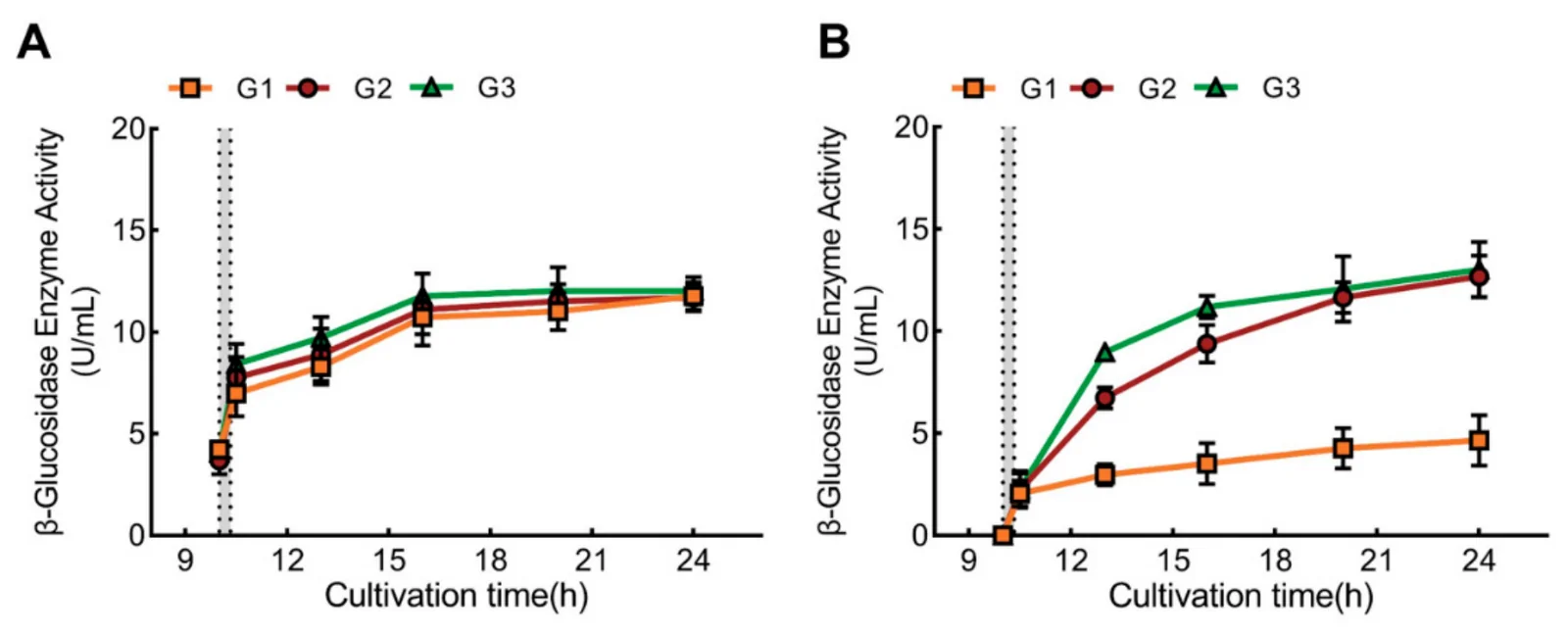
Partitioned β-glucosidase activity: (**A**) intracellular activity and (**B**) extracellular activity. G1, conventional fermentation; G2, low-dose UAF; G3, high-dose UAF. The grey vertical dashed line indicates the time point of ultrasound application (10 h). Intracellular activity rose synchronously across groups from 10.5 h without significant inter-group differences, whereas extracellular activity diverged from approximately 10.5 h onward, reaching 3.52 ± 0.82, 9.38 ± 0.76, and 11.19 ± 0.45 U/mL at 16 h in G1, G2, and G3, respectively. This pattern is consistent with increased extracellular β-glucosidase availability and may reflect externalisation/leakage associated with altered cell-envelope integrity; it is not direct evidence of ultrasound-induced de novo enzyme synthesis.

Partitioned β-glucosidase activity profiles are presented in Figure 6. Across the 10–24 h window, intracellular β-glucosidase activity rose synchronously in all three inoculated groups (G1, G2, and G3) without significant differences among treatments (Figure 6A).

In contrast, extracellular β-glucosidase activity diverged among groups following ultrasound treatment at 10 h (Figure 6B). From 10.5 h onward, extracellular activities in G2 and G3 remained consistently higher than in G1, reaching 3.52 ± 0.82 U/mL in G1, 9.38 ± 0.76 U/mL in G2, and 11.19 ± 0.45 U/mL in G3 at 16 h. Notably, the dose-dependent elevation of extracellular *β*-glucosidase activity occurred without significant shifts in intracellular activity. This selective increase indicates enhanced membrane permeability or localised envelope disruption, which may permit passive enzyme efflux from a vulnerable cell subpopulation. Our findings instead support a physical accessibility mechanism: calibrated UAF facilitates the release of pre-existing intracellular *β*-glucosidase and enhances enzyme–substrate contact within the fermentation matrix [15].

#### 3.7.2. Microscopy Evidence Relevant to Cell-Envelope Permeability and Matrix Disruption

**Figure 7:**
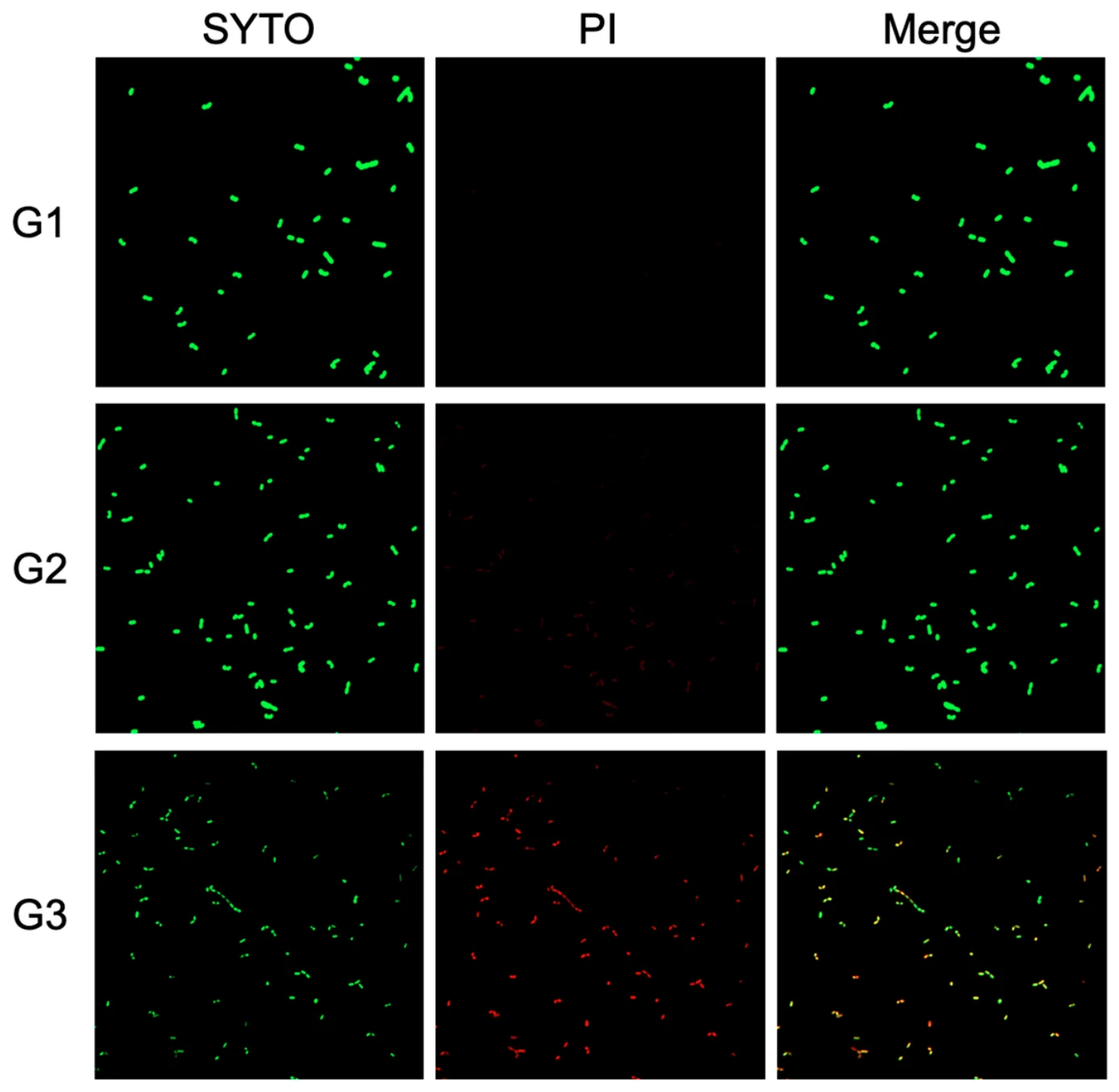
Representative CLSM images of *L. plantarum* cell-envelope staining after ultrasound treatment. Cells were dual-stained with SYTO9 (green) and propidium iodide (PI; red). G1 was predominantly SYTO9-positive and PI-negative, whereas G3 showed more PI-accessible cells in the representative fields. Images represent qualitative observations from a single experimental run without multi-replicate, multi-field PI quantification, consistent with altered cell-envelope integrity but not establishing a specific permeabilisation mechanism. All images were acquired at 40× magnification.

Qualitative microscopic observations of both *L. plantarum* cell-envelope staining and *Astragalus* matrix morphology further support this interfacial interpretation. Representative CLSM fields showed predominantly SYTO9-positive, PI-negative cells in G1 and more PI-accessible red-fluorescent cells in G3 following ultrasound intervention (Figure 7). 

**Figure 8:**
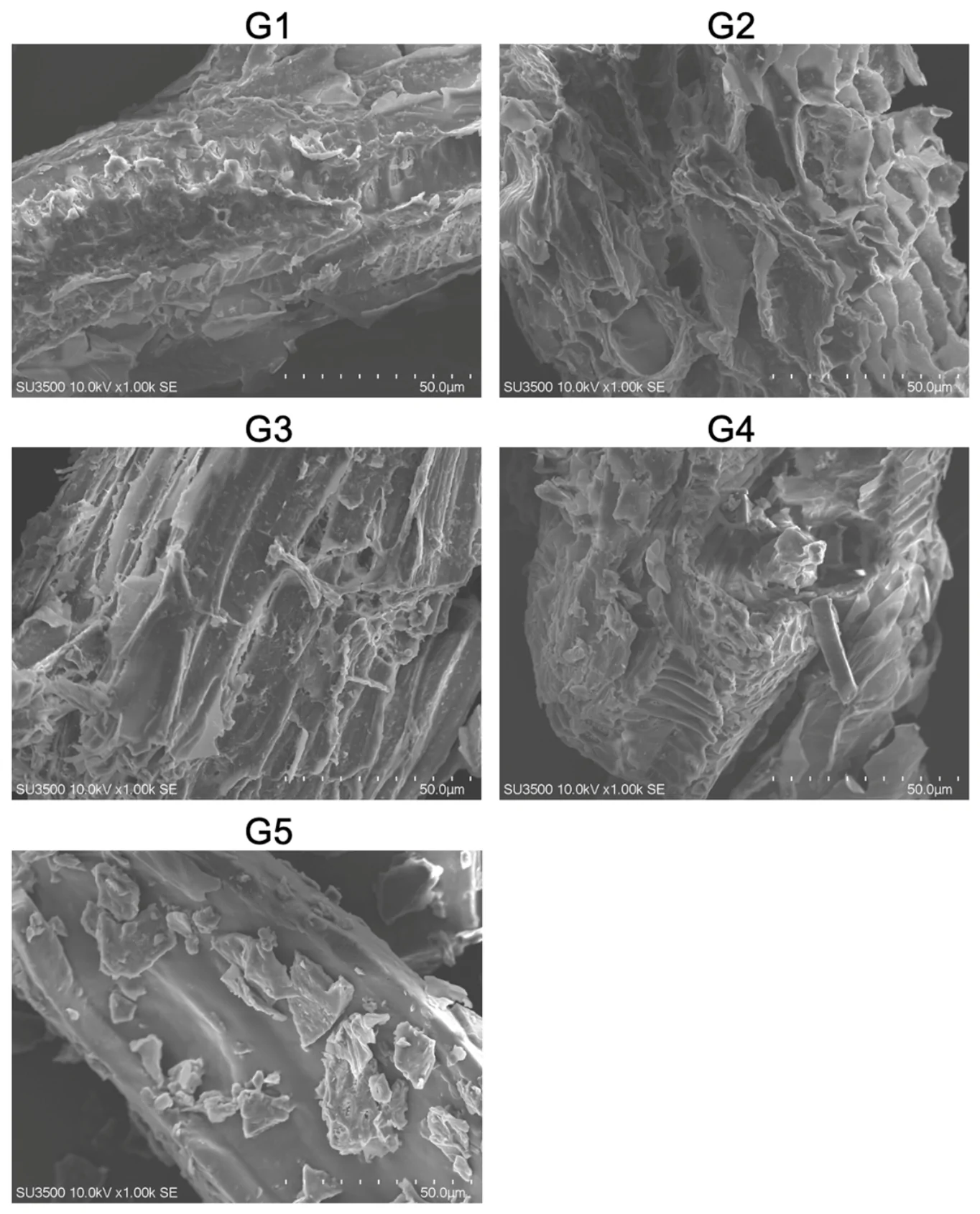
SEM images of *Astragalus* matrix morphology after 18 h of fermentation. (G1) Compact and relatively smooth surface architecture. (G3) Surface fragmentation, grooves, and pore structures after high-dose UAF. Magnification, 1000×. These qualitative morphological differences are consistent with enhanced matrix disruption, although substrate accessibility was not measured directly.

Concurrently, SEM imaging of the *Astragalus* matrix at 18 h revealed distinct substrate-side morphological differences between groups (Figure 8). Whereas the matrix recovered from G1 displayed a relatively compact and smooth surface architecture, G3 exhibited surface fragmentation, grooves, and pore structures. These morphological differences may be relevant to enzyme–substrate contact [34].

Taken together, the increased extracellular β-glucosidase availability on the microbial side and the physical disruption of the *Astragalus* matrix on the substrate side provide a plausible dual-interface explanation for the earlier attainment of the conventional 24 h aglycone concentration.

From a bioprocess perspective, a single 20 min sonication at 10 h minimized total acoustic exposure, though net energy efficiency remains to be quantified. Translating these kinetic improvements beyond the 30 mL scale to probe-type, multi-transducer, or continuous flow-through configurations will demand renewed calorimetric calibration and rigorous control over acoustic field uniformity.

## 4. Conclusions

Under high-dose ultrasound-assisted fermentation (86.05 ± 4.03 W/L), aglycone concentrations reached 99.7% of the 24 h benchmark within 18 h, with a 50% higher mean formation rate during the 10–18 h window. The sterile ultrasound control showed no detectable direct ultrasonic hydrolysis of the target glucosides, indicating that the enhanced aglycone formation was primarily associated with fermentation-related processes. At the 18 h time-matched sampling point, UAF also enhanced *in vitro* antioxidant capacities, digestive-enzyme inhibitory activities, and intestinal-phase aglycone recovery. Enzyme activity profiles and morphological observations further support a dual mechanism involving enhanced extracellular β-glucosidase release and physical disruption of the *Astragalus* matrix. Collectively, these findings demonstrate the potential of UAF as an effective strategy to accelerate aglycone formation and improve the functional properties of fermented *Astragalus*.

## Figures and Tables

**Table 1:** Water-based calorimetric estimates for the fermentation vessel configuration (nominal bath power-density settings of 50 and 100 W/L; 30 mL sample, 100 mL Erlenmeyer flask, 40 kHz bath, 600 s sonication).

Nominal Bath Power Density (W/L)	Δ*T* over 600 s (°C)	Water-Calibrated Power Estimate (W)	Water-Calibrated Power-Density Estimate (W/L)
50	6, 7, 7	1.40 ± 0.12	46.51 ± 4.03
100	13, 12, 12	2.58 ± 0.12	86.05 ± 4.03

**Table 2:** Kinetic parameters of isoflavone aglycone formation during conventional and calibrated ultrasound-assisted fermentation of *Astragalus membranaceus* (mean ± SD, *n* = 3).

Kinetic Parameter	G1	G2	G3
Mean formation rate, 10–18 h (μg g^−1^ h^−1^) ^1^			
Calycosin	6.17 ± 1.71	7.20 ± 0.98	9.40 ± 1.71
Formononetin	3.76 ± 0.69	3.65 ± 0.12	5.47 ± 1.43
Total aglycones	9.93 ± 2.37	10.85 ± 1.05	14.87 ± 3.11
AUC_10–24h_ (total aglycones, μg h g^−1^) ^2^	8525.1 ± 12.5	8697.3 ± 66.5	8831.7 ± 63.6
Total aglycone concentration at 24 h (μg g^−1^)	665.6 ± 7.2	665.5 ± 12.6	669.6 ± 14.8
Key comparison			
G3@18 h/G1@24 h (%) ^3^	/	/	99.7 ± 2.4%
18→24 h increase (%) ^4^	+7.3 ± 2.0%	+4.8 ± 1.8%	+0.9 ± 4.1%

Notes: ^1^ Mean formation rate over 10–18 h = (*C*_18h_ − *C*_10h_)/8 h, calculated for each replicate and then averaged. ^2^ Area under the total-aglycone concentration–time curve from 10 to 24 h (trapezoidal rule), calculated for each replicate and then averaged. ^3^ Total aglycone concentration of G3 at 18 h expressed as a percentage of G1 at 24 h (per-replicate ratio, mean ± SD). ^4^ Percentage increase in total aglycone concentration from 18 to 24 h, calculated for each replicate and then averaged.

## Data Availability

Data will be made available on reasonable request.

## Supplementary Materials

The following supporting information can be downloaded at: https://www.mdpi.com/article/10.3390/foods15213909/s1, Figure S1. Baseline fermentation kinetics of *L. plantarum* in the *Astragalus* matrix without ultrasound intervention. Viable cell counts and pH were monitored over 0–28 h. The logarithmic-phase growth and synchronous pH decline supported the selection of 10 h as the ultrasound intervention window. Figure S2. Optimisation of ultrasound timing. EG1, conventional fermentation without ultrasound; EG2, ultrasound pretreatment of sterile *Astragalus* medium prior to inoculation; EG3, ultrasound intervention during the logarithmic phase. Active-compound content and β-glucosidase activity were highest in EG3, supporting logarithmic-phase intervention over medium pretreatment under the tested conditions. Figure S3. Historical HPLC profiles from a β-glucosidase incubation assay. Because the archived records do not uniquely identify the reaction substrate, this figure is provided for reference only and is not used to infer substrate specificity or reaction mechanism in the present study.
